# The practice of commissioning healthcare from a private provider: learning from an in-depth case study

**DOI:** 10.1186/1472-6963-13-S1-S4

**Published:** 2013-05-24

**Authors:** Naomi Chambers, Rod Sheaff, Ann Mahon, Richard Byng, Russell Mannion, Nigel Charles, Mark Exworthy, Sue Llewellyn

**Affiliations:** 1 Manchester Business School, University of Manchester, Booth Street West, Manchester, M15 6PB, UK; 2 University of Plymouth, Drake Circus, Plymouth, PL4 8AA, UK; 3 Peninsula College of Medicine and Dentistry, John Bull Building, Research Way, Plymouth, Devon, PL6 8BU, UK; 4 University of Birmingham, Edgbaston, Birmingham, West Midlands, B15 2TT, UK; 5 Royal Holloway, University of London, Egham Hill, Egham, Surrey, TW20 0EX, UK

## Abstract

**Background:**

The direction of health service policy in England is for more diversification in the design, commissioning and provision of health care services. The case study which is the subject of this paper was selected specifically because of the partnering with a private sector organisation to manage whole system redesign of primary care and to support the commissioning of services for people with long term conditions at risk of unplanned hospital admissions and associated service provision activities. The case study forms part of a larger Department of Health funded project on the practice of commissioning which aims to find the best means of achieving a balance between monitoring and control on the one hand, and flexibility and innovation on the other, and to find out what modes of commissioning are most effective in different circumstances and for different services.

**Methods:**

A single case study method was adopted to explore multiple perspectives of the complexities and uniqueness of a public-private partnership referred to as the “Livewell project”. 10 single depth interviews were carried out with key informants across the GP practices, the PCT and the private provider involved in the initiative.

**Results:**

The main themes arising from single depth interviews with the case study participants include a particular understanding about the concept of commissioning in the context of primary care, ambitions for primary care redesign, the importance of key roles and strong relationships, issues around the adoption and spread of innovation, and the impact of the current changes to commissioning arrangements. The findings identified a close and high trust relationship between GPs (the commissioners) and the private commissioning support and provider firm. The antecedents to the contract for the project being signed indicated the importance of leveraging external contacts and influence (resource dependency theory).

**Conclusions:**

The study has surfaced issues around innovation adoption in the healthcare context. The case identifies ‘negotiated order’, managerial performance of providers and disciplinary control as three media of power used in combination by commissioners. The case lends support for stewardship and resource dependency governance theories as explanations of the underpinning conditions for effective commissioning in certain circumstances within a quasi marketised healthcare system.

## Background

The direction of health service policy in England is for more diversification in health care services provided by newly configured NHS services, voluntary, local government and corporate sector organisations; and for greater diversity in commissioning organisations. The NHS will be required to ensure that effective, efficient, patient-centred and safe services are commissioned but without stifling flexibility and innovation [1,2]. Public-private partnerships (PPPs) are being encouraged. These take many forms, including outsourcing, public finance initiatives (PFI) and strategic partnering, but essentially arise from make or buy decisions that governments face to deliver societal goals. The relationships between state and private actors is argued as having resulted in the phenomenon of hybridity in which the players develop both public and private orientations and interests [3]. In contrast with the US and some parts of Europe, PPPs are a relatively new phenomenon in the UK. Outside the health sector, there has been a drive to view commissioning and procurement away from a merely purchasing activity focussing on price and time-to-market, towards a more strategic process, which comprises finding, developing and leveraging external resources to help achieve the goals of the firm [4,5]. An important question is how to find the best strategic means of achieving a balance between control and flexibility, and to find out what modes of commissioning are most effective in different circumstances and for different services.

We use two main theoretical frames in order to understand the processes of effective healthcare commissioning. The first focuses on the use of power, and the second focuses on corporate governance practices for setting direction and exerting control.

The first theoretical framework that underpins this case study and the main associated study on the practice of commissioning is a contingency theory of modes of commissioning [6]. This theory explores how commissioning practice interacts with co-existing hierarchical and networked governance [7,8]. Governance is an exercise of power. Therborn’s conceptualisation of power has been applied and adapted to healthcare commissioning to identify governance mechanisms that commissioners may employ in the practice of commissioning [9,10]. These governance mechanisms are conceptualised as media of power. Six distinct media of power that a commissioner may exercise in their relationships with providers have been identified. These are: negotiated order, provider competition, financial incentives, ideological and disciplinary control, juridical governance and managerial performance. Each medium of power is described in the context of commissioner-provider relationships in Table 1.

**Table 1:** Six media of power exercised in commissioner-provider relationships

Media of power	Description and sources
Medium 1 – Negotiated order (relationality)	Conflicts are managed to produce a “negotiated order” [11]. The emphasis is on relationality [12]. Negotiated order is characterised by explicit or tacit mutually agreed arrangements between commissioners and providers about their involvement in and responsibilities for commissioning. Such mutually agreed arrangement might relate to information sharing and the division of labour, for example.

Medium 2 – Provider competition (contestability)	This medium of power relates to commissioners’ attempts to manage competition between providers [13]. Three key features of this include the criteria for selecting providers, the range of providers and monopsonisation - buyer-side monopoly [14]

Medium 3 – Financial incentives	Commissioners may employ a number of financial incentives to influence provider behaviours. These may relate to units of payment [15], timing of payment, terms and conditions, bonuses, penalties and exemptions [16].

Medium 4 – Ideological and disciplinary control (professional and political ideologies)	Ideological and disciplinary controls through discursive “orders” may be employed by commissioners in their negotiations [17-19]. For example these might relate to technical or scientific knowledge (such as evidence based practice), occupational ethos and norms of conduct, political and economic belief-systems and appeals to higher managerial and political authority such as “targets”, regulators or managerial elite.

Medium 5 – Juridical governance (contracts and law)	Legal and regulatory mechanisms may be used by commissioners in various ways [20]. This medium of power might refer to contract specifications, the use or threat of coercive enforcement of contracts or legal rights and the use of arbitration.

Medium 6 – Managerial performance of commissioning (managerial performance repertoires)	Commissioners have a range of managerial mechanisms and resources to draw on when negotiating with providers [21]. These include decisions about which stakeholders are involved, the role of external supporting bodies, scrutiny of provider performance, prevailing models of commissioning and delegation of commissioning roles and responsibilities.

Combinations of different media of power produce distinct “modes of commissioning”. The nature of such modes is contingent upon the context within which decisions are made, such as the speciality under review, geography and the institutional features of the commissioning organisation. Some media of power may reinforce each other whilst others may conflict (elements of juridical control and negotiated order for example). This is consistent with Osborne and Brown’s [22] view that inherent contradictions in the policy context (such as simultaneous centralisation and decentralisation) and different institutional and temporal contexts means that various forces play out in different ways. By combining different *media of power* to identify specific *modes of commissioning* the theoretical framework developed here offers a means of both recognising and reconciling any inherent contradictions in the relationship between commissioners and providers, in the context where they are created and sustained.

This framework is outlined in more detail in a parallel article in this edition of BMC HSR, drawing from material collected in the same Department of Health funded research study on the practice of commissioning, which contrasts the dominant modes of commissioning used in Germany with those found in England [6].

In examining the process of commissioning in this case study, we also employ a second theoretical frame drawing from a corporate governance perspective to examine the conditions for effective commissioning governance. Jensen and Meckling [23] emphasise the generality of the agency problem of stakeholders being able to exert adequate control, both at all levels of management and also across different types of organisations, including non-profit organisations, government corporations and cooperatives in corporate and contract governance. Also taking an agency view, Berle and Means argue that problems of exerting adequate control exist as a result of different or opposing sets of interests between managers and shareholders, public administrators and taxpayers, and, in healthcare, between commissioners and providers [24]. Stewardship theory and resource dependency theory propose different explanations based, respectively, on assumptions around a joint endeavour between the parties in a united view around securing better outcomes, and the need for boundary spanning to secure investment and enhanced reputation [25,26]. Stakeholder governance is concerned with the creation of long term public value and an organisation purpose which satisfices a wider community interest. With the exception of agency theory, these theories also resonate with the negotiated order as a medium of power described above, and also with the use of PPPs for strategic partnering purposes. Each main corporate governance theory and associated governance practices is outlined in Table 2.

**Table 2:** Conjunction of main corporate governance theories and practices

THEORIES	PRACTICE	
	FOCUS	DYNAMICS

**Agency:** Control of management; managers and owners/stakeholders have different interests; [23,24]	Supervision of management Risk minimisation & compliance Monitoring of performance against targets and objectives	High challenge Controlling Critical style in order to achieve goals

**Stewardship**: Joint endeavour with management; managers and owners/stakeholders have same interests [25]	Strategic thinking Emphasis on improvements & excellence in performance & use of resources	Appreciative style to achieve goals Collaborative

**Resource Dependency**: Leveraging of external expertise and influence as organisation success dependent upon fit with external environment [26]	Policy formulation Horizon scanning Inward investment of resource and reputation	Predominantly external orientation Management through influence

**Stakeholder**: Mirroring community and society served to ensure that organisation serves its mission and purpose [28,29]	Supervision of management Monitoring of performance against targets and objectives Creation of long term public value	System/ society focussed orientation Consensual Mindful of sectional or political interests

(Source: [27])

Our research questions therefore relate to these two theoretical frames: which media of power outlined above are combined to enact modes of healthcare commissioning, and to what effect, and which corporate governance theories have most utility in understanding the underpinning practices of healthcare commissioning.

## Methods

To answer these questions, the Livewell (not its real name) project was chosen. Yin outlined the potential for analytic generalisation from the use of case study methodology, for example that one or more cases are typical of other situations or examples, and thus lead the way to theory building [30]. Taking a somewhat different epistemological stance, the single case study approach is advocated by Simons [31,32] as a way of getting beneath the surface of policy implementation, helping to challenge accepted thinking and current forms of knowing and to reconstruct understanding. Case study explores multiple perspectives of the complexity and uniqueness of a project or programme in a ‘real life’ context [31][p21]. The argument runs that this alternative way of seeing by focussing on the uniqueness of the case produces uncertainty, disruption and new insights. And the paradox of case study is that *‘...by studying the uniqueness of the particular*, *we come to understand the universal...’ *[31][ p231]. Taking this a step further, it can be argued that in studying the individual case, unique insights also arise from the perspectives of each participant who are part of the case, all of whom are the street level bureaucrats [33] making their own sense of government policy as they implement it. Greenhalgh and colleagues argue that the rich description made possible by in-depth case study is the key to understanding the dynamic complexities in social life and particularly in healthcare; further they suggest that the study of ‘language games’ (i.e. contextualised talk and action) of multiple stakeholders offer important insights leading to *heuristic generalisation* (i.e. what is really going on here?) [34].

Policy related to healthcare commissioning is in a state of flux, transition and uncertainty. Coupling this with the highly contextualised nature of the topic, and the political, social, economic and professional complexity of the system that it resides within (see also Allen [35] and Porter et al [36] in this special issue); a case study approach appears especially valuable. The elicitation of unique features and the study of language games which are central to Simons and Greenhalgh’s perspectives appear to us to be particularly germane in tackling questions of the actual use of power and control and the choices made in the mechanisms of governance deployed in the practice of commissioning of health care.

Livewell is a single in-depth case study of a public-private partnership. It was selected as a stand-alone mini case study site because of government policy to encourage private sector involvement in commissioning. In this instance, it involves the partnering with a private sector organisation, as co-commissioner and provider, to aid whole system redesign in primary care and to support the commissioning of services for people with long term conditions at risk of unplanned hospital admissions. It can be characterised as a strategic partnering form of public private partnership longer term collaboration, in contrast with other forms identified by Skelcher such as PFI, contracting out, franchising, and joint venture [3]). The programme is described in more detail in Additional file 1.

10 single depth interviews, audio recorded and transcribed, were carried out with key informants involved in the practices in the commissioning and management of the Livewell initiative and with the private partner lead. The interviewees were selected on the basis of the widest possible range of professional backgrounds and roles in the initiative. The topics covered in the interviews are outlined in Additional file 2. These were worked up to elicit the media of power and corporate governance practices in use in commissioning in this case. The identity of our informants was kept confidential and findings are reported anonymously. Informants were consulted as to whether they would like copy of the transcript of their interviews. This offer was taken up by one: there was no revision suggested to the transcript as a result. One other respondent was keen to have his/her job title more precisely recorded in the report and in publications. Documentary evidence was also gathered from key documents and evaluations (for example the evaluation of phase 1 of the programme; patient information sheets) and the researcher also attended programme management meetings. The project has ethical approval from the NHS Research Ethics committee for the South West of England. Governance arrangements of the project are organised by the Peninsula Clinical Research Network.

The case study was analysed using a common analytic framework including fields reflecting the media of power and the governance practices described above. To check whether the framework omitted important data patterns, we also inductively coded the qualitative data to add new themes to the analytic framework [37]. The findings were developed using a thematic analysis and a grounded theory approach [38], as well as a coding framework based on the six media of power and corporate governance theories. The main themes that we identified and outlined in the findings were: the participants’ understanding and use of the term ‘commissioning’; challenges in primary care; ambitions for the project; roles and relationships; impact, adoption and spread of innovation; and impact of changes in commissioning arrangements. In the discussion section, we then proceed to highlight the dominant media of power deployed, and the main corporate governance practices in use in this case study, before drawing further provisional conclusions for theory and practice.

## Results

Interviewees came from four of the five practices and include 4 GPs [GP1, GP2, GP3, GP4], 2 advanced nurse practitioners [ANP1, ANP2] 1 practice manager [PM], 1 business manager [BM], the private provider lead [PPL] and a Primary Care Trust chief executive [CE]. To preserve anonymity, the job titles have in some cases been slightly altered to more generic roles.

### Understanding and use of the term ‘commissioning’

Not surprisingly, given the full title of the Livewell programme which includes the term ‘service redesign’, respondents did not particularly like the term ‘commissioning’:

*‘.....We wouldn’t use the word commissioning at all. And*, *actually*, *we think it’s over-laboured in the UK. Far too much time and attention is given to commissioning and far too little time and attention is given to services.... we really want to see a sea change. We want to demonstrate what services look like when they’re good.’* [BM].

The term ‘service redesign’ was preferred by all respondents, apart from the PCT chief executive. This redesign project was developed, designed, and signed off by patients, carers, care givers, and other stakeholders. One respondent asserted that the term commissioning contains an inherent assumption that there is certainty about what is required, although in reality there may not be certainty or good evidence about what will work.

The second point made was about the use of integrated care as an organising principle. This was illustrated by thinking on services improvement in the programme around a fictional woman called ‘Maisie’ who is a carer for her mother who has dementia and who has her own health problems. Third, there was support for the notion that effective commissioning delivers better quality for a lower cost. Fourth, there was emphasis on effective ‘*hands on’* micro-commissioning (the doing of service redesign) at the frontline, including the use of analytics, financial modelling, and making use of the experiences of users and care-givers. Finally there was a warning about the need not to underestimate the length of time required to achieve change and the emotional labour of the commissioning work:

*‘....It* [commissioning] *will make a difference provided everybody has got a lot of time*, *the clinician sitting there has got a lot of time to do all the job. Even if you understand what needs to be done*, *to change the system it takes a very*, *very long time. Sometimes the secondary care they work in their own way*, *they may not agree with you and sometimes even if they agree with you*, *to change them it takes many years and sometimes* (*dividing the pot*) *that causes a lot of problems....’.* [GP3]

### Challenges and choices in primary care

Respondents described significant problems in motivating patients to choose healthier life styles and to look after themselves better in such a deprived area, where the patients have so many other economic and social problems to contend with. They also reported pressure by patients for referral to specialists in a search for medical solutions, and other struggles to contain care outside hospital. The reality of the practice of primary care was exemplified by the comment below from a GP from one practice:

*‘......Why do we send to hospital? I’m not talking about somebody with a really complicated history*, *I’m talking demented lady*, *living at home*, *had a fall at home*, *when you go [to their] home*, *no relatives*, *nobody to give them medicine. They didn’t take their medicine for two days so you ring the local social worker – no answer - and if you look for a nurse*, *there’s hardly any community service now. So at that time only one person will answer your telephone call*, *that is the hospital. Nobody else*, *that is the only hope for you*, *that the person goes to hospital and then at least they’ll be better for that and somebody will feed them*, *somebody will give them medicine and do some basic tests. But if we [had] certain things available.... we wouldn’t need the hospital’* [GP3]

In this context, respondents reported six main ambitions for the project: to seek beneficial changes to people’s health, help patients to take greater control, change how services are used, secure greater self-management and management in primary care, deliver strategic thinking on whole system redesign, and to make savings. The chief executive from the PCT put it like this:

*‘What we wanted to see was the evidence*, *captured elsewhere*, *of how changing the way that care is delivered can result in some really beneficial changes to patients’ health*, *on the one hand*, *but also change the way that patients used healthcare facilities. So*, *we were working with [name of private sector provider] to look at the changes to health for people with long term conditions when they were given support in a different way.... And in particular*, *we were looking at morbidity*, *[reductions in the] number of hospital admissions that might result from people being able to self-manage themselves in a more effective way. But also being able to receive that support primarily from primary care rather than actually to have to go into hospital..... [and] I guess we saw the project and investment we put in has potential to lead into longer term savings because*, *of course*, *with PBR we’re paying for every time that people with long term conditions enter into a hospital environment*, *and we knew*, *of course*, *all the evidence shows us is that people with long term conditions are those that occupy the majority of hospital beds....’* [PCT CE]

There was not absolute consensus. One GP [GP2] was doubtful that the telephone care management service would diminish A & E attendances although it might reduce hospital admissions. Another GP [GP3] thought it would help with improving patient understanding of their long term condition, modify lifestyle behaviours affecting their health as well as halting the ‘*disease process’*, reducing hospital admissions, and lengths of stay. Related ambitions were to change perceptions in the minds of patients about long term conditions having a wholly external locus of control, to challenge folklore about the need for A & E attendance to provide confirmation of the severity of the health problem, and to tackle some of the mental health issues that often go along with having more than one long term condition. A powerful image was conjured by one respondent in describing the importance of giving *‘armbands’* to the *‘weak swimmers’* - which is what the telephone support service has done.

Although the foreseen effect was to reduce use of secondary care services, the point was made that the primary aim of the project was to improve care for patients.

### Roles and relationships

Four aspects to roles and relationships came through strongly in the interviews. First, the primacy of the practices, and in particular the GPs, in driving the service redesign project; second, the close relationship with the private sector provider; third, the spirit of co-design and co-production with the private provider; and, fourth, the history of a positive relationship developed with the Primary Care Trust (PCT) the local body responsible for healthcare commissioning at the time of the study.

One of the reported characteristics of this project was that the level of commissioning was spearheaded at the practice level rather than at the PCT. This meant ownership was at that level rather than the PCT ‘selling’ the initiative. Our participants indicated that the project was led, in the main, by the doctors rather than by nurses or other staff from the practices. It was doctors who decided who should be excluded from invitations to participate in the service (for example because a patient had a terminal illness). Nurse practitioners and practice nurses attended briefing sessions and were involved in the development of the group consultation service. Of the primary health care team, community nurses from the local NHS community service organisation run by the primary care trust probably had least exposure to the project. There was also no evidence that the local hospital were involved in the design. Practice managers were involved in liaising with the private sector partner particularly around access to patient records. There was at least one example of ideas from the practice staff however that had not appeared to have percolated up either to the practice or further. Patient group members were invited to quarterly forums and gave views on the text of letters to be sent to patients and on survey questionnaires.

One example of collaboration was the very close co-design of the telephonic care management service with the private sector partner. After the risk stratification tool had been utilised, GPs in all 5 practices checked their lists of patients for suitability for invitation to telephone support service. As the private provider lead said:

‘*To us it’s just patients on a list. We don’t know who’s hard of hearing*, *who has compliance issues*, *who is even possibly in day care during the day*, *so we do rely on them really being able to go through that list at a high level and just being able to say: look this person’s really not appropriate. And sometimes our risk model will miss patients that really could benefit*, *so if they do come across somebody that they think would be perfect for the programme we can invite them into the programme and get their data. So I think their [GP] involvement there has been really instrumental in that initial patient determination’* [PPL].

The private sector partner had been initially brought in at the first phase of the Livewell initiative in a strategic partnership, without a formal tender: ‘*an agreement in principle that we wanted to work together’* [BM].

They were engaged for their expertise and fresh outlook to offer overall programme management support, provide the in-depth practice profiles on activity and costs, be the clinical lead on the telephonic care management service, and to be partners in strategic thinking on whole system redesign (the ‘think-tank’ work). The private sector partner was described by respondents as prepared, organised, interested, patient and responsive:

*‘The [Private sector partner] were the only ones who wanted to work with little people like us and develop a product’* [GP4].

The overall relationship was described as close, with a strong rapport, working as one team, learning together and a desire for a win-win between both parties. Latterly, with the advent of the second phase of the Livewell initiative, which was a single action tender and recognising that both parties owned some intellectual property, a more transactional tone was reported to be coming to the fore at times with more of a focus on KPIs (key performance indicators). The second generation practices reported a courteous but more distant relationship with the commissioning support firm, indicating in their interviews that the first phase of the project had done much of the trialling and they had been able to take advantage of that.

On the whole, the spirit of co-design, co-production and learning together came through very strongly. A trial and error approach was adopted as it was agreed that a US model without adaptations to suit the multicultural local population in this inner city area in England would not work. For example, not only was the initial determination of the choice of patients to receive the telephone support service co-delivered in collaboration with the practices, but the system for feedback and referral back to practices of patients by the telephone care management nurses was also redesigned, following some difficulties with conveying uncorroborated patient viewpoints about perceived deficiencies in GP surgery services. One respondent, in relation to his/her role as a GP commissioner, commented favourably on being able to work with a healthcare provider *‘from the inside’* and a preparedness on the part of the private sector provider *‘to work with the little people’* in contrast with the more distant relationships experienced with providers in normal circumstances.

There was a history over some years of the general medical practices developing positive relationships with the PCT. More than one respondent described humorously how the PCT had been *‘drip tortured’* into investing in the initiative. Unusually, the PCT (by the admission of the PCT chief executive as well as other respondents) encouraged and supported, rather than commissioned this project. But the investment was not easily won and required tenacity:

*‘It’s....about creating some of those relationships and the relationship building...... Sometimes I don’t agree with what people say and you don’t agree with their mode of working*, *you just have to stick with it. It requires staying power. And also for them to know that you are*, *we are the organisational memory here*, *I’ve been round so many times’* [GP4]

### Impact, adoption and spread of innovation

In terms of evidence, respondents indicated that, for the time being, the positive impact in the Livewell programme was largely experienced by very positive patient feedback from the telephonic care management service rather than from statistical evidence about a decrease in A & E attendances and admissions. There are plans in place to report to practices changes to biometrics of affected patients, such as blood pressure, levels of blood sugar, weight, breathlessness and so on, recognising that changes are likely to take at least a year before they are seen.

Second phase practices acknowledged that much of the hard work had been done in the first phase and that although they had not been involved from the beginning, they benefitted from that. They gave the impression of being more casual in their motives for joining the project. One also reported that having been invited to join the second phase, they decided ‘*why not...give it a try’?* [GP2].

Respondents reported issues with followership, embedding innovation such as risk stratification in general practice and the challenge of scaling up. The second phase practices were more likely to have either the clinicians or the practice manager closely involved in the project rather than both. There were some problems expressed with practices signing up but not necessarily wholly committed. One practice indicated that they felt ‘a *little in the dark....and not near the [*lead practice] *level [*PM 1]. Not all were convinced of the value of group consultations. In parallel with this, those considered to be the innovators or entrepreneurs also reported experiencing a degree of mistrust at times from their wider peer group.

On the other hand, the involvement of a wider group of practices had tested the project conception and design, and resulted for example in the programme embracing the challenge of managing the psychological aspects of long term physical health conditions. One of the practices reported the wider use within the PCT of the idea of the telephone care management service to ring their very elderly patients during the winter period to check that they were ok. Another example was the potential use of the practice profiles developed by the private sector partner to probe cost and value of obstetrics services (particularly for pre term monitoring admissions) and the possibility of primary care based fracture reviews.

### Destabilisation: the impact of changes in commissioning arrangements

The influences of health reform were felt in three respects. First, there was an acknowledgement of the changing health policy and political context and not least a change of government and the consequences of this for the private sector partner, as the market for private providers had shifted. Second, it was noted that the project was an invest-to-save project that crossed between the old and new NHS commissioning regimes. This meant that there was some rupture in long term relationships, the new commissioning architecture was complex, Third, PCT staff were leaving so that data extraction process had been delayed by up to nine months, and there was now an absence of the proactive involvement, support and encouragement from the original funder.

## Discussion

In this mode of commissioning, around strategic long term partnering with a private sector commissioning support organisation and provider, we note that the three dominant media of power were relational: first, the development and maintenance of a negotiated order (Power medium 1, Table 1) through agreed information sharing, division of labour and close collaboration; second, in order to secure funding in the first place, tenacity in an appeal to the scientific evidence underpinning the project (Power medium 4, Table 1) and, third, managerial performance of the procurement process, in its widest sense, with the service supplier being as proactive in this regard as the procurer (the practices) which we can also term ‘provider-led commissioning’ (Power medium 6, Table 1).

We were particularly interested in this notion of ‘closeness’ between provider and commissioner – terms themselves not used by the participants to describe themselves, preferring the term ‘partner’. There is here a possibility of getting ‘too close’, that is without sufficient challenge, or in corporate governance terms, focussing on a joint endeavour or stewardship approach, which itself has been found to contain that potential hazard [27]. There may also be a contingency perspective at play, that is an understanding about the stage in the commissioning or service improvement cycle when this was entirely appropriate to achieve the objectives of both parties and in particular when this a new kind of initiative for both parties. We have considered also whether this closeness was indicative of more widely found behaviours of service providers from the private sector with their customers or clients, in contrast to providers in the public sector who may more generally keep their distance from the commissioners. Cousins and Spekman describe a climate of change in private sector supply chain management thinking, which would endorse this, signalling a move away from arms-length relationships towards a mutual obligation-based approach between suppliers and purchasers [5].

We noted that additional media of power were added to the commissioning repertoire, on occasion, for example contract enforcement (power medium 5, Table 1) through the use of the monitoring of adherence to key performance indicators, and as well as appeal to scientific evidence, the use of ideological control (power medium 4, Table 1) through the foregrounding and espousal of preferred policies such as integrated care and risk stratification for active case management.

From a governance perspective, we noted the dominance of a spirit of high trust and close professional alignment between the parties to achieve the negotiated order, which tends to support the stewardship theory of the joint endeavour, as opposed to the agency theory predicated on the two parties having different sets of interests and therefore low trust (see Table 2). In the project set-up phase, we also noted the significance of boundary spanning to leverage external influence and resource which mirrors the resource dependency corporate governance theory. The term ‘drip torture’ was used (by themselves) to describe the tenacity with which the leadership of the programme sought financial support, courteously stalking the PCT over a sustained period until their request was acceded to.

Some further preliminary conclusions can be drawn from this study, to be tested and refined with follow-up phone calls with participants, with their permission. The first one is in relation to the practice of micro-commissioning which provides a particular insight into the managerial performance of procurement (power medium 6) in the healthcare context. As a preliminary, the general practice participants were averse to the term commissioning itself, with *service redesign* or a *new offer* being preferred terminology. One of the most striking observations of this project is the closeness of the two parties in service design and development and the acknowledgement that this is a lengthy, uncertain, iterative and labour intensive process to gain traction, ownership, change and measurable impact. Provider-led commissioning was also permitted: where the commissioning support firm had very specific expertise and experience in service design and provision (for example in analytics and in the development of the telephonic care management service), the commissioner (the GPs) let them lead on the design of those services.

Primary care clinicians described themselves as *‘little people’*, more than once. This perception of relative powerlessness in the overall healthcare system and the measures taken to mitigate this state of affairs as observed in this case study can aid our understanding of effective commissioning when there are perceived significant power differentials.

Some spin-off consequences of employing a different kind of health care provider with fresh ideas were described. The challenge of scaling up, of embedding innovation across 5 practices rather than within just one practice was noted. This reflects a level of risk aversion in the NHS and the problem of a somewhat ossified healthcare system. It confirms a prevailing organisation culture which is more inclined to incremental rather than to disruptive innovation.

### Practical lessons for commissioners in the English NHS and other countries

We think that there are a number of practical lessons for commissioners in the NHS in England and for healthcare design, commissioning and procurement in other countries, which relate to our original research questions around media of power and alternative approaches to governance:

1. There has to be an acknowledgement that the very term ‘commissioning’ is problematic and can be a ‘turn off’, particularly for the clinicians who may be at the heart of the process, either as commissioners or as deliverers of care. It is a peculiarly English term. Alternative more acceptable terminology could be considered and may include: service redesign, development, improvement, procurement, purchasing; with clinical commissioning groups becoming the ‘design house’.

2. The case study has shown the benefits of a mode of commissioning characterised by closeness (‘deep commissioning’ or ‘micro commissioning’) between commissioners and providers in service development, recognising the often tortuous path to securing actual improvements for patients. This closeness to commissioners is something that providers too may wish to consider. We do not see this as being necessarily a characteristic of working with a private provider of health care or with commissioning support services. It may however be that in this case the private provider was more innately customer focussed than public sector providers might be. There are also to suspect that the closeness is connected with the nature of complex long-term care and common, undifferentiated complex conditions, for example multi-morbidity in frail elderly often with social marginalisation reasons (see the papers by Porter et al [36], and Sheaff et al [6] in this special issue). We do not yet know what measurable benefits have been achieved by this programme for patients but the high levels of trust and the degree of embeddedness of provider with commissioner stands out and points to a very productive mode of commissioning. More research in this area to understand the circumstances when this approach would be particularly beneficial is called for.

3. The research study is predicated on the existence of a media of power commissioning repertoire, which makes use, alternately, of relational approaches, contracting levers, markets and competition, ideological disciplines, financial incentives and robust management of procurement. The project under investigation favoured the first, particularly in the start-up phase, enhancing a creative spirit of joint enterprise. Commissioners may wish to consider selecting more deliberately which approach suits particular circumstances.

4. We would suggest that the underpinning governance principles for a commissioning repertoire could be amplified to include agency, stewardship, resource dependency and stakeholder frames. In practice, this means that commissioners may select, and switch between, a low trust and high challenge approach with providers (for example when there are concerns about service weakness or failure), a high trust and high challenge approach for generating innovative ideas for service improvement, or an externally focussed boundary spanning approach (building relationships with key stakeholders in the system) for example to enhance reputation, win resources and inward investment.

5. Identification, support and backing for the relatively rare radical thinkers and innovators in the NHS in England, particularly in primary care, would be helpful to speed up the pace of change. In parallel, these radical innovators may wish to consider the value of relationship building and the use of carefully constructed selective long term restatement of their ideas with key stakeholders, described as *‘drip torture’* in this study, as a mechanism for getting their views heard.

6.The consequences of structural reform on the commissioning side of the NHS in England may be a temporary slowing down of innovation and implementation of service improvement due to ruptures in long term relationships in circumstances where these, given the emphasis on negotiated order and stewardship governance, have begun to be productive. There are warnings here for other countries considering embarking on structural healthcare reform. The argument that there is currently a misplaced focus on structural rather than service reform in the NHS to solve efficiency and effectiveness problems has been made elsewhere [39]. This case study offers further evidence of this and spells out the consequences, particularly in relation to the rate of being able to improve services for vulnerable patients.

## Competing interests

None.

## Authors' contributions

NC carried out the fieldwork and data analysis for the single case study reported in this manuscript and drafted this manuscript. All authors contributed to this manuscript and to the design, fieldwork and analysis of the main study on commissioning practice, funded by the Department of Health.

## Supplementary Material

Additional file 1 The Livewell Programme Click here for file

Additional file 2 Livewell Case Study Interview Topics Click here for file

## Declarations

Publication of this supplement was funded by the Nuffield Trust.

This article has been published as part of *BMC Health Services Research* Volume 13 Supplement 1, 2013: The Limits of Market-based Reforms. The full contents of the supplement are available online at http://www.biomedcentral.com/bmchealthservres/supplements/13/S1.
