# Supplementary material for: The practice of commissioning healthcare from a private provider: learning from an in-depth case study
Source: BMC Health Serv Res. 2013 May 24;13(Suppl 1):S4. doi: 10.1186/1472-6963-13-S1-S4 (PMC3663660; doi:10.1186/1472-6963-13-S1-S4)
Supplement: Additional file 1 — The Livewell Programme [file 1472-6963-13-S1-S4-S1.docx]

**The Livewell Programme**

**The area:** Livewell is an area of the West Midlands characterised by high levels of deprivation, communities with a high proportion of people from diverse minority ethnic backgrounds, poor levels of general health and high levels of chronic disease prevalence. In common with other areas, the health economy is challenged by high and increasing levels of A & E attendances and urgent care admissions. 32 different first languages are spoken in the community.

**Hospital utilisation in the area:**  Across the 5 practices in the Livewell programme, 32% of urgently admitted patients stay in hospital for less than 24 hours; 60% of A & E attendances result in patients being discharged of whom 40% go home without treatment; there is a large degree of variation in urgent admissions, A & E attendances and outpatient referrals between the five practices. In one of the practices, where a more depth analysis has been undertaken, 54% of spend has been estimated to be on urgent care in contrast with inpatient elective (16%), day cases (20%) and maternity (10%).

**The programme objectives**:

- the provision of improved care to patients
- better results in providing services to hard-to-reach groups
- enhanced self care
- workforce development
- new ways of accessing services
- reduction of secondary care use
- a proactive risk-based approach to screening to target care
- improved value for money

**Patient and front line staff involvement:** Driven by the doctors there was also a series of events held with staff to shape the programme strategy and redesign. Patient participation groups were actively involved on an ongoing basis in design and in offering detailed feedback. Users and care givers took up structured opportunities for engagement.

**Phases 1 & 2**: Phase 1 of the programme started in 2008 and covered one practice, of 9,500 patients. Programme management support was secured from a private sector healthcare commissioning support provider who also delivered the telephone care management service. Phase 2 of the initiative, which was conceived in 2010, and sponsored by the local primary care trust (PCT) as a fund-to-save initiative (approx £500k), comprised 5 practices covering 50,000 patients and was supported by the same private sector firm. Redesign of care included an initiative to run group consultations, a think-tank, and a telephone care management service. It is this last, in phase 2 of the project, that is the principal focus of this case study.
