# Supplementary material for: The practice of commissioning healthcare from a private provider: learning from an in-depth case study
Source: BMC Health Serv Res. 2013 May 24;13(Suppl 1):S4. doi: 10.1186/1472-6963-13-S1-S4 (PMC3663660; doi:10.1186/1472-6963-13-S1-S4)
Supplement: Additional file 2 — Livewell Case Study Interview Topics [file 1472-6963-13-S1-S4-S2.docx]

**Livewell Case Study Interview Topics**

1. What do you think commissioning in health care is about?
2. What do you understand by the term ‘commissioning’?
3. What are the most effective tools in commissioning in health care in your view?
4. When does innovation happen in primary care?
5. What are the aims of the Livewell initiative?
6. Why did you get involved?
7. How did you secure the funding?
8. How was the private sector partner selected?
9. How is the contract with the private sector partner managed?
10. What are the benefits to patients of this initiative?
11. What will happen next?
